# The Brocchieri Styptic

**Published:** 1846-03

**Authors:** 


*The Brocchieri Styptic.*—Prof. Mott, of New York, in a late clinical lecture, thus alluded to this article, and its discoverer, M. Brocchieri:

"I knew M. Brocchieri when I was in Paris; he is an uneducated man, and a perfect charlatan. When his discovery was made known in Paris, it created some stir; and I made several experiments with it, in connection with several other gentlemen, one of whom was engaged in the preparation of the watar. The subjects of the experiments were strong and healthy sheep, upon whose carotids we operated, and we found that its power to stop hemorrhage was next to nothing, and where the bleeding was arrested it was principally from the pressure made by the large quantities of lint, with which the wound was filled. Therefore, I say, as the result of my experience, that the styptic powers of this preparation are not to be relied upon, for a moment: that it is infinitely less useful than an infusion of rhatany, or tannan, and that it can never take the place of needles and ligature.

The other qualities that have been ascribed to it, of curing diseases. and arresting hæmoptysis, are equally non-existant.—*N. Y. Med. and Surg. Reporter.*

